# Dose-Related Effects of Cannabidiol on Inflammatory and Redox Responses, Bacterial Dissemination, and Multiorgan Injury in CLP-Induced Septic Rats

**DOI:** 10.3390/ph19101649

**Published:** 2026-10-19

**Authors:** Ufuk Ülker, Bülent Bayraktar, Tünay Karan, Çağrı Çağlar Sinmez, Emre Tüfekçi, Esra Bilici, Umut Alpman, Gökşad Cemil Kotan, Mustafa Yeni

**Affiliations:** 1 Department of Veterinary Microbiology, Faculty of Veterinary Medicine, Yozgat Bozok University, 66100 Yozgat, Türkiye; ufuk.ulker@bozok.edu.tr; 2 Department of Physiotherapy and Rehabilitation, Faculty of Health Sciences, Bayburt University, 69000 Bayburt, Türkiye; bulentbayraktar@bayburt.edu.tr; 3 Department of Genetics, Faculty of Veterinary Medicine, Yozgat Bozok University, 66200 Yozgat, Türkiye; tunay.karan@bozok.edu.tr; 4 Department of History of Veterinary Medicine and Deontology, Faculty of Veterinary Medicine, Erciyes University, 38039 Kayseri, Türkiye; cagrisinmez@erciyes.edu.tr; 5 Department of Wild Animal Diseases, Faculty of Veterinary Medicine, Erciyes University, 38039 Kayseri, Türkiye; emretufekci@erciyes.edu.tr; 6 Laboratory and Veterinary Health Program, Eşme Vocational School, Uşak University, 64600 Uşak, Türkiye; esra.bilici@usak.edu.tr; 7 Department of Surgery, Faculty of Veterinary Medicine, Erciyes University, 38039 Kayseri, Türkiye; umutalpman@erciyes.edu.tr; 8 Laboratory and Veterinary Health Program, Department of Veterinary Medicine, Alaca Avni Çelik Vocational School, Hitit University, 19600 Çorum, Türkiye; goksadcemilkotan@hitit.edu.tr; 9 Department of General Surgery, Erzurum Regional Training and Research Hospital, 25240 Erzurum, Türkiye

**Keywords:** cannabidiol, sepsis, cecal ligation and puncture, oxidative stress, cytokines, adipokines, bacterial translocation, multiorgan injury, histopathology

## Abstract

**Background:** Sepsis couples dysregulated inflammation, oxidative imbalance, microbial dissemination, and multiorgan injury. Cannabidiol (CBD) has anti-inflammatory and redox-modulating properties, but its dose-related effects in polymicrobial sepsis, particularly alongside culture-recoverable bacterial burden, remain incompletely characterized. **Methods:** Forty-eight male Wistar rats were randomized to Control, Sham, Untreated CLP, or CLP plus CBD at 2.5, 5, or 25 mg/kg (*n* = 8/group). CBD or vehicle was administered intraperitoneally after cecal ligation and puncture (CLP). Inflammatory, immunometabolic, neurotrophic, oxidative, biochemical, microbiological, and histopathological outcomes were evaluated at 10 h. **Results:** Untreated CLP increased pro-inflammatory cytokines, asprosin, resistin, oxidative-stress indices, organ-injury biomarkers, and reported culture-recoverable bacterial burdens, while decreasing IL-10, apelin, BDNF, TAS, and immunoreactive SOD, CAT, and GPx concentrations (all omnibus *p* < 0.001). CBD was associated with progressive improvement across most systemic outcomes, and 25 mg/kg produced the most consistent overall response. Histopathological lesion scores were greatest in Untreated CLP animals and generally lower in CBD-treated groups; however, tissue-level changes were not uniformly dose-ordered across every organ and lesion domain. **Conclusions:** Within this acute exploratory model, CBD exposure was associated with coordinated improvements across host-response, redox, biochemical, and morphological domains. The microbial summaries suggested lower recoverable burdens, but require animal-level verification of non-detect handling. These findings support pharmacokinetic, safety, mechanistic, survival, and prospectively powered confirmatory studies; they do not establish direct antibacterial activity or a clinically applicable dose.

## 1. Introduction

Sepsis is life-threatening organ dysfunction caused by a dysregulated host response to infection and remains biologically heterogeneous despite advances in supportive care [1,2,3,4]. The early syndrome integrates pathogen sensing, endothelial and microvascular dysfunction, mitochondrial stress, immunothrombosis, and maladaptive innate and adaptive immune responses [3,4,5,6]. These processes uncouple tissue oxygen delivery from utilization, disrupt epithelial barriers, and promote acute kidney, hepatic, pulmonary, cardiovascular, and cerebral injury [5,6,7,8,9]. Cecal ligation and puncture (CLP) reproduces an evolving polymicrobial focus and is therefore widely used to interrogate this network in vivo, although model severity depends strongly on ligation length, needle size, puncture number, resuscitation, analgesia, and sampling time [10,11].

Oxidative stress is not merely a by-product of inflammation in sepsis. Excess mitochondrial and enzymatic reactive oxygen species amplify lipid peroxidation, redox-sensitive inflammatory signaling, endothelial permeability, and cell death, whereas depletion or inactivation of superoxide dismutase (SOD), catalase (CAT), glutathione peroxidase (GPx), and low-molecular-weight antioxidants limits host resilience [12,13,14,15]. Malondialdehyde (MDA), total oxidant status (TOS), total antioxidant status (TAS), and the oxidative stress index (OSI) provide complementary information on this imbalance. Parallel measurements of TNF-α, IL-1β, IL-6, and IL-10 are needed because sepsis involves simultaneous pro- and counter-inflammatory programs rather than a simple cytokine excess [4,6].

The metabolic and neurotrophic response is also integral to sepsis biology. Apelin/APJ signaling supports cardiovascular and endothelial homeostasis and has shown protective effects in experimental septic myocardial and pulmonary injury [16,17,18,19]. Resistin is linked to macrophage-driven inflammation and sepsis severity [20,21,22], whereas asprosin integrates glucogenic, insulin-resistance, and inflammatory stress responses [23,24,25]. Brain-derived neurotrophic factor (BDNF) reflects neuronal-survival and plasticity pathways and has been associated with outcomes in critical illness [26,27]. These mediators were selected because CBD has been reported to exert vascular, metabolic, immunomodulatory, and neuroprotective effects; their joint assessment could therefore indicate whether any treatment-associated change extended beyond conventional cytokine and redox endpoints. Their measurement was exploratory and was not intended to demonstrate direct CBD target engagement.

Cannabidiol (CBD) is a non-intoxicating phytocannabinoid selected for this study because it combines antioxidant, immunomodulatory, endothelial-protective, and neuroprotective properties within a single intervention, whereas many candidate adjuncts target only one component of the septic response [28,29,30,31,32,33]. CBD can influence adenosine signaling, PPARγ-associated responses, redox-sensitive transcription, inflammasome activity, and cellular oxidative balance; however, these mechanisms are context dependent and were not measured directly here. In rats subjected to CLP-induced sepsis, Cassol et al. reported reduced oxidative damage in selected organs, improved later cognitive performance, and reduced mortality [34]. In a separate experimental pneumococcal meningitis model, Barichello et al. reported that CBD attenuated inflammatory responses and reduced cognitive impairment [35]. These findings made CBD a biologically plausible candidate for testing whether early inflammatory control can coexist with lower, rather than higher, culture-recoverable bacterial dissemination.

We tested 2.5, 5, and 25 mg/kg CBD after CLP to explore whether early inflammatory and redox changes were accompanied by lower culture-recoverable bacterial burden. Inflammatory cytokines (TNF-α, IL-1β, IL-6, and IL-10) and redox outcomes (MDA, TOS, TAS, OSI, and immunoreactive SOD, CAT, and GPx) constituted the main scientific focus. Immunometabolic and neurotrophic mediators, biochemical organ-injury markers, bacterial burdens, and histopathology were complementary exploratory outcomes. This distinction describes the scientific objectives; it is not a prospectively registered hierarchy of confirmatory primary and secondary endpoints. All statistical findings are exploratory. The single 10 h endpoint cannot establish an optimal dose, long-term survival benefit, direct antibacterial activity, or a molecular mechanism.

## 2. Results

### 2.1. Organ Function and Injury Biomarkers

CLP produced a broad biochemical phenotype of early multiorgan injury (Table 1). Relative to Control, Untreated CLP increased AST approximately threefold, ALT 3.5-fold, total bilirubin 3.8-fold, creatinine fourfold, BUN 3.7-fold, NGAL 5.3-fold, KIM-1 4.3-fold, lung MPO 4.8-fold, and S100B 4.6-fold. CBD was associated with graded improvement at 2.5, 5, and 25 mg/kg. At 25 mg/kg, AST, ALT, bilirubin, creatinine, BUN, NGAL, KIM-1, MPO, and S100B were substantially lower than in Untreated CLP and close to the non-septic range. All omnibus comparisons were significant (*p* < 0.001). Tukey-adjusted comparisons showed that each CBD-treated group differed from Untreated CLP for every endpoint in Table 1 (*p* < 0.05); shared lowercase letters identify nonsignificant pairwise differences.

### 2.2. Cytokine, Immunometabolic, and Neurotrophic Responses

The Untreated CLP group showed a coordinated pro-inflammatory and metabolic-stress response: TNF-α, IL-1β, IL-6, asprosin, and resistin increased, whereas IL-10, apelin, and BDNF decreased (Table 2). The direction of change was progressively reversed across CBD doses. At 25 mg/kg, TNF-α, IL-1β, and IL-6 were reduced by 78.8%, 80.1%, and 80.2%, respectively, relative to Untreated CLP, while IL-10 recovered from 28.6 ± 3.1 to 64.2 ± 6.1 pg/mL. Apelin and BDNF rose toward control values, whereas asprosin and resistin declined. Together, these changes showed a coordinated dose-related pattern across inflammatory, immunometabolic, and neurotrophic endpoints. Each CBD-treated group differed from Untreated CLP for every endpoint in Table 2 (Tukey-adjusted *p* < 0.05).

### 2.3. Oxidative Stress and Antioxidant Defense

CLP increased MDA, TOS, and OSI while decreasing TAS and the normalized immunoreactive levels of SOD, CAT, and GPx (Table 3). These changes were directionally consistent with higher oxidative burden, lower total antioxidant status, and reduced immunoreactive antioxidant-enzyme protein levels. CBD produced a dose-ordered response across all seven endpoints. At 25 mg/kg, MDA was 67.3% lower and TOS 64.6% lower than in Untreated CLP, whereas TAS, SOD, CAT, and GPx recovered to 92.6%, 90.6%, 92.0%, and 92.8% of Control values, respectively. OSI fell from 3.98 ± 0.38 in CLP to 0.63 ± 0.07. Each CBD-treated group differed from Untreated CLP for every endpoint in Table 3 (Tukey-adjusted *p* < 0.05).

### 2.4. Recoverable Bacterial Burden

Untreated CLP generated high local, blood, and tissue culture-recoverable burdens (Table 4). Counts were lower in a dose-ordered pattern in every sampled matrix. At 25 mg/kg, aerobic and anaerobic peritoneal counts were 4.70 and 4.74 log_10_ units lower than Untreated CLP, and blood burden was 4.03 log_10_ units lower. These in vivo findings do not demonstrate direct antibacterial activity. Across the culture endpoints reported in Table 4, CBD-treated groups consistently showed lower bacterial burdens than the Untreated CLP group. The corresponding Tukey-adjusted comparisons are presented in Table 4; given the presence of culture-negative observations, these inferential findings were interpreted as exploratory.

### 2.5. Exploratory Histopathology

Control sections had no recorded degenerative/necrotic lesions in liver, kidney, spleen, heart, or lung (Table 5). Mild splenic lymphoid-follicle hyperplasia and occasional vascular hyperemia were recorded in several Control cases. Sham sections showed predominantly low-grade hepatic and renal degeneration, splenic follicular hyperplasia, and mild vascular changes (Table 5).

Untreated CLP animals showed a broad pattern of histopathological injury across the examined organs (Table 5). Degenerative and necrotic changes were most prominent in the kidney (54.3 ± 8.8%), lung (54.5 ± 9.0%), and liver (51.8 ± 10.3%), while pulmonary inflammation reached 54.5 ± 9.6%. Vascular alterations were likewise evident, particularly in the lung (52.0 ± 10.0%) and liver (44.8 ± 9.4%). Taken together, these findings indicate a substantial multiorgan lesion burden in untreated septic animals and provide the morphological reference against which the CBD-treated groups can be descriptively interpreted.

#### 2.5.1. Histopathological Lesion Patterns in CBD-Treated Cohorts

In the CLP + CBD 2.5 and 5 mg/kg groups, histopathological lesions remained evident but were generally less pronounced than those observed in Untreated CLP animals. In the 2.5 mg/kg group, the highest mean scores were recorded for hepatic degeneration (27.3 ± 16.9%) and renal degeneration (21.3 ± 8.6%), while pulmonary inflammation and vascular changes averaged 13.9 ± 10.3% and 10.6 ± 8.8%, respectively (Table 5). In the 5 mg/kg group, hepatic degeneration remained the most prominent lesion (30.0 ± 16.0%), whereas renal degeneration was lower (13.1 ± 15.3%); pulmonary inflammatory and vascular scores were 13.9 ± 12.4% and 14.8 ± 13.8%, respectively (Table 5). Overall, the 2.5 and 5 mg/kg groups showed a broadly similar histopathological burden, although the distribution of lesions varied across organs and lesion categories.

In the CLP + CBD 25 mg/kg group, lesion scores were generally lower across the examined organs. Mean hepatic and renal degeneration scores were 18.9 ± 12.2% and 12.3 ± 8.6%, respectively, while pulmonary inflammation averaged 11.1 ± 5.7%. Vascular alterations were comparatively limited, with mean liver and lung scores of 7.3 ± 3.4% and 4.4 ± 4.2%, respectively (Table 5). Although these findings suggest a generally lower lesion-score profile at 25 mg/kg, the pattern was not uniformly dose-ordered across every organ and lesion domain and should therefore be interpreted descriptively.

#### 2.5.2. Interpretation of the Exploratory Histopathological Findings

Group-level histopathological summaries are presented in Table 5. Untreated CLP animals showed the greatest overall lesion burden, whereas CBD-treated groups generally had lower scores. Differences varied across organs and domains and were not uniformly dose-ordered. These descriptive findings complement the systemic measurements without establishing histological efficacy.

**Figure 1:**
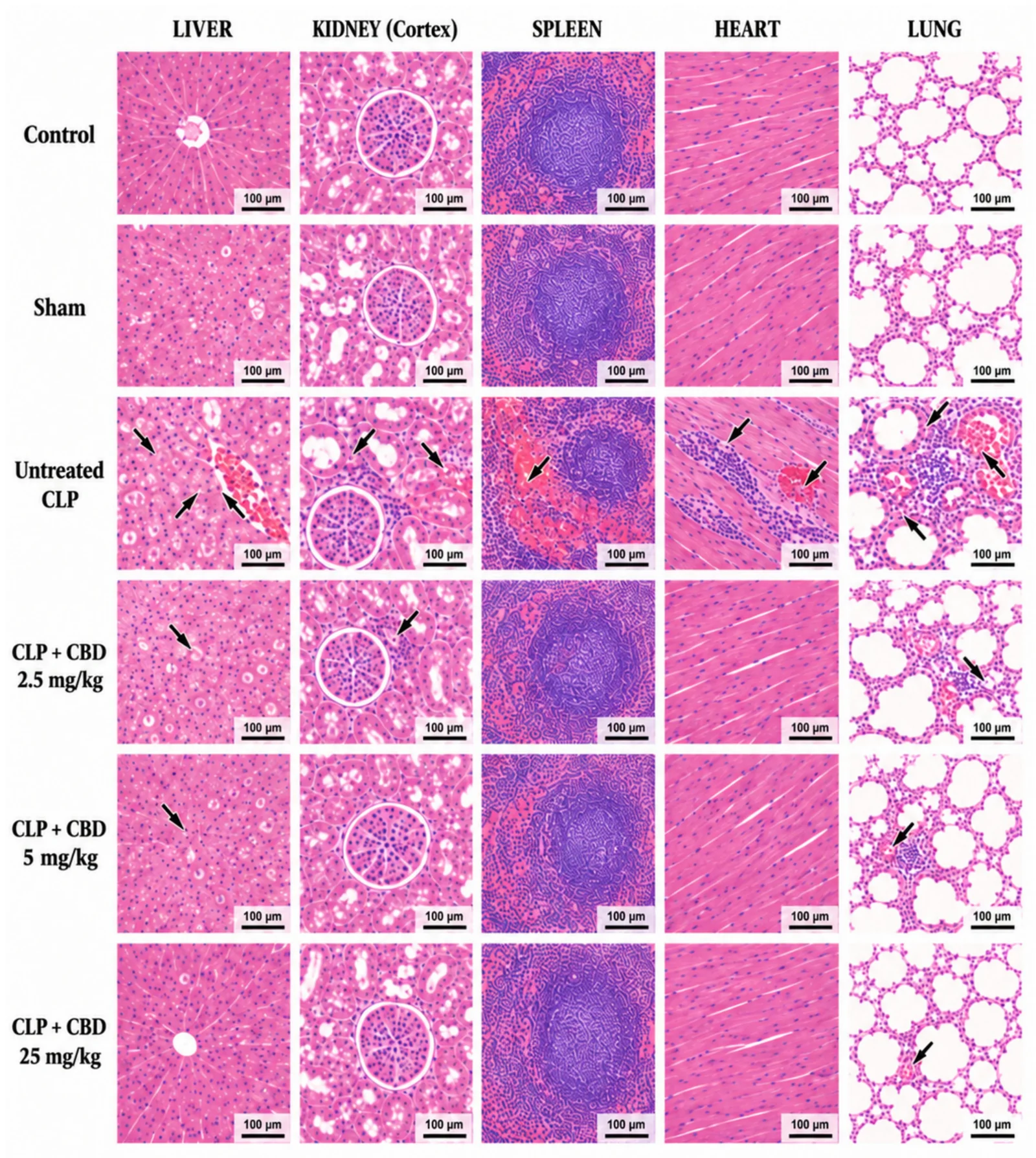
Representative hematoxylin and eosin (H&E)-stained sections of the liver, kidney cortex, spleen, heart, and lung from Control, Sham, Untreated CLP, CLP + CBD 2.5 mg/kg, CLP + CBD 5 mg/kg, and CLP + CBD 25 mg/kg groups. Untreated CLP sections show more prominent degenerative/necrotic, inflammatory, and vascular alterations, whereas CBD-treated sections generally show less prominent histopathological alterations. Black arrows indicate representative histopathological alterations: hydropic degeneration and sinusoidal congestion in liver; tubular degeneration and luminal proteinaceous casts in kidney cortex; red pulp hyperemia in spleen; myocardial fiber degeneration and interstitial hemorrhage in heart; and alveolar septal thickening with inflammatory cell infiltration in lung. Scale bars = 100 µm.

**Figure 2:**
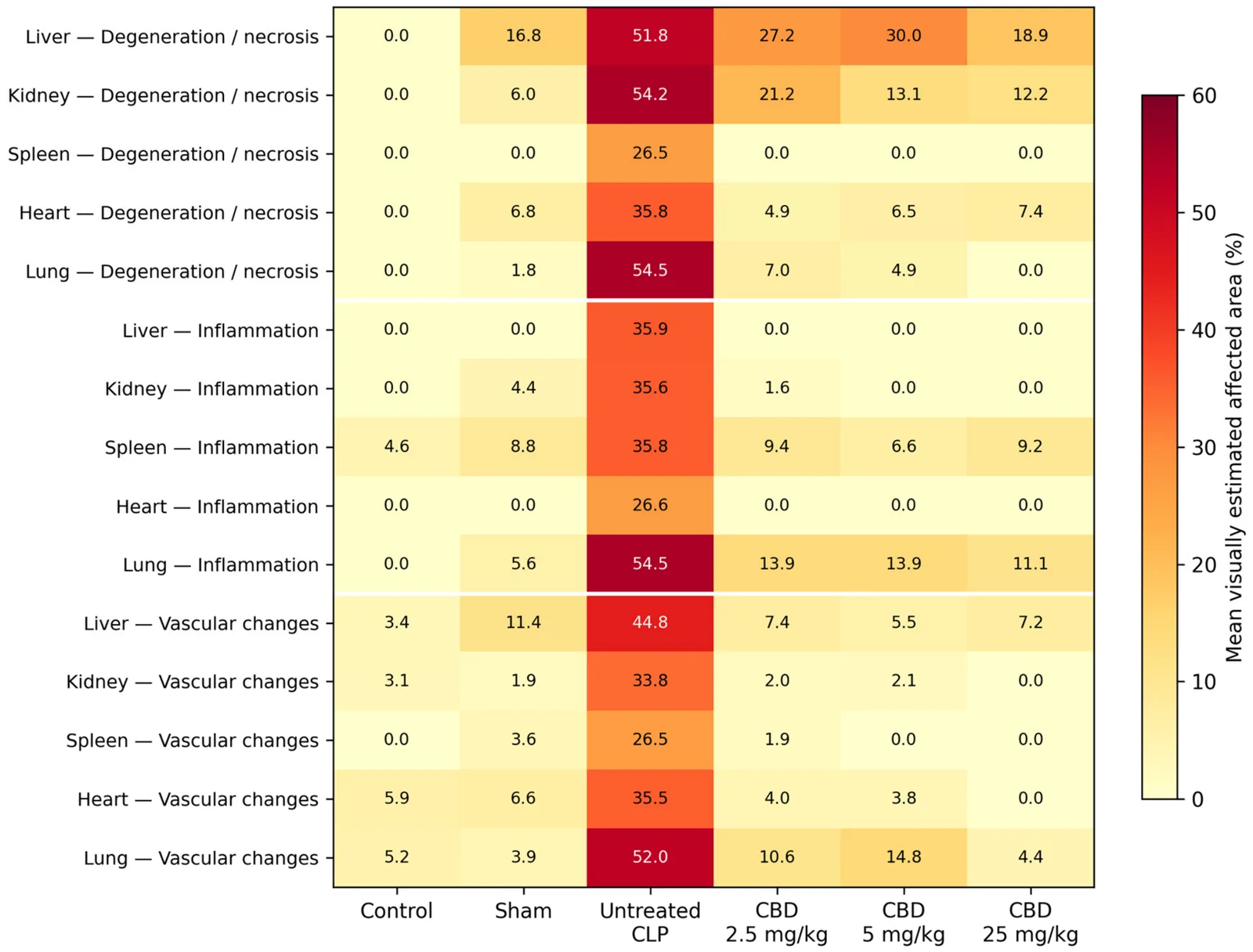
Descriptive heatmap of mean histopathological lesion scores (% affected area), *n* = 8 animals/group. Each cell is an animal-level group mean, not a significance result. CBD, cannabidiol; CLP, cecal ligation and puncture.

The systemic and histopathological observations were directionally concordant but do not establish causal mediation. Representative H&E sections are retained in Figure 1, while Figure 2 shows the group mean lesion percentages. The original panel image is reproduced without resampling or alteration; interpretation of particular arrowed lesions and scale calibration requires the source microscopy records.

## 3. Discussion

Within this acute dose-ranging experiment, CBD exposure was associated with a coherent response across inflammatory, redox, organ-injury, immunometabolic, neurotrophic, and microbiological domains. The 25 mg/kg group showed the most consistent overall separation from Untreated CLP, whereas the histopathological pattern was directionally supportive but not uniformly dose-ordered across every organ and lesion category. The principal pharmacological observation is therefore the concordance of responses across independent outcome classes without an accompanying increase in culture-recoverable bacterial burden, rather than evidence of complete rescue or proof of a single mechanism. This extends experimental work showing CBD-associated modulation of oxidative injury, inflammatory responses, cognition, and survival in CLP sepsis and related inflammatory models [34,35]. Nevertheless, the present evidence is limited to a single early endpoint and does not establish receptor-level target engagement, direct antimicrobial activity, an optimal dose, survival benefit, or clinical efficacy.

The inflammatory findings are consistent with the experimental literature describing CBD-associated modulation of pro-inflammatory cytokines and oxidative injury across CLP sepsis and other inflammatory models [28,29,30,31,32,33,34,35]. Adenosine signaling, PPARγ-associated responses, NF-κB-related transcription, and inflammasome regulation are plausible explanations derived from external studies, not pathways confirmed by our experiment. Their relevance here therefore remains hypothetical pending target-engagement measurements. The increase in IL-10 alongside lower pro-inflammatory mediators suggests early rebalancing rather than uniform cytokine suppression, although the meaning of IL-10 is time-dependent and cannot be extrapolated beyond 10 h.

The measured cytokine, redox, organ-injury, and microbial changes suggest coupled host-response differences. They do not identify causal mediation. Adenosine, PPARγ, NF-κB, Nrf2/HO-1, inflammasome, mitochondrial, and glutathione pathways are hypotheses from external studies and require direct perturbation and target-engagement experiments [28,29,30,31,32,33,34,35,36,37,38,39,40,41,42].

Lower MDA and TOS together with higher TAS and immunoreactive SOD, CAT, and GPx were consistent with earlier experimental reports [29,31,32,33,34,39,40,41]. The immunoassays measured antioxidant-enzyme protein concentrations, not catalytic activities. Direct redox-flux and enzyme-activity assays are needed to determine functional antioxidant effects.

The renal response was characterized by concordant changes in creatinine and BUN as functional indices and in NGAL and KIM-1 as early tubular-injury markers. The concordant decrease in all four reduces the likelihood that the apparent benefit reflects only altered filtration or hydration. Hepatic injury showed the same pattern across AST, ALT, and bilirubin. Lower lung MPO suggests reduced neutrophil-associated pulmonary inflammation, whereas lower S100B and higher BDNF values are compatible with attenuation of early neuroinflammatory and barrier-related injury. These findings align with the known multiorgan nature of CLP and with studies showing that CBD is associated with attenuation of endothelial, cardiac, renal, and neural injury in inflammatory, oxidative, and experimental sepsis models [31,34,35,39,40,41,42]. Because AST is not liver-specific and serum S100B is not a stand-alone measure of encephalopathy, the multimarker context is essential.

The immunometabolic findings extend the current literature by showing coordinated changes in apelin, resistin, and asprosin within the same acute CLP experiment. Apelin fell sharply after CLP and increased across the CBD-treated groups. Experimental apelin/APJ activation can support hemodynamics, endothelial integrity, and myocardial function in sepsis [16,17,18,19], so higher apelin levels may reflect improved cardiovascular-metabolic adaptation. Resistin behaved in the opposite direction, consistent with its pro-inflammatory biology and clinical association with sepsis severity and mortality [20,21,22]. Asprosin also increased with CLP and declined with CBD. Recent clinical studies have proposed circulating asprosin as a diagnostic or severity-related marker in sepsis [24,25], while the mechanistic literature connects asprosin to hepatic glucose output, insulin resistance, and inflammatory stress [23]. The parallel changes in apelin, resistin, and asprosin suggest that the treatment-associated response extended beyond conventional cytokine changes to immunometabolic mediators. Because feeding state, glucose, insulin, and body composition were not measured, causal metabolic interpretation remains provisional.

BDNF was markedly depressed by CLP and approached control values at the highest CBD dose. Lower circulating BDNF levels have been associated with mortality in critically ill patients, including those without clinically evident brain dysfunction [26,27]. A previous CLP study linked CBD treatment to improved later cognitive outcomes [34], while related experimental work in pneumococcal meningitis reported attenuation of cognitive impairment [35]. A more recent CLP investigation also reported effects of CBD on long-term brain alterations after sepsis [42]. Our 10 h BDNF and S100B results provide an early biochemical bridge to that literature. They cannot demonstrate preserved cognition because behavioral neurocognitive testing was not performed, and peripheral BDNF is influenced by platelet handling and preanalytical conditions.

Apelin, resistin, asprosin, and BDNF are exploratory circulating biomarkers. Feeding state, glucose and insulin responses, platelet release, sample handling, and systemic illness severity may affect their concentrations. Standardized serum processing reduces some technical variation but cannot isolate these biological influences or establish direct CBD action on the corresponding pathways. A potentially important result was the dose-ordered reduction in culture-recoverable bacteria from peritoneal fluid, blood, and organs. Although CBD has shown antimicrobial activity in dedicated in vitro studies [43,44,45], no minimum inhibitory concentration, time-kill, tissue-exposure, or ex vivo killing experiment was performed here; the present result therefore must not be described as direct antibacterial activity. A more conservative interpretation is that CBD exposure coincided with less recoverable dissemination during the 10 h window, potentially through indirect effects on barrier integrity, perfusion, phagocyte function, or the host inflammatory environment. Antibiotic-controlled and culture-independent studies are required to separate these alternatives. The zero-substitution rule for non-detects can exaggerate or attenuate apparent differences. In particular, below-limit group means must not be treated as measured subthreshold bacterial concentrations. Without verified animal-level CFU data, the robustness of the microbial comparisons to alternative detection-limit handling remains unresolved.

Additional experimental sepsis studies provide complementary support. Maayah et al. reported attenuation of systemic inflammation and cardiac and renal injury in an LPS-induced sepsis model [44], while Erkiliç and Bayraktar reported higher serum BDNF and lower cTnI levels following CBD treatment in a 10 h CLP rat model [45]. Untreated CLP animals had the greatest descriptive lesion burden, particularly in the liver, kidney, and lung, consistent with multiorgan injury in experimental sepsis [10,11,36]. CBD-treated animals generally had lower scores, which is compatible with previous experimental CBD reports [34,39,40,41]. The nonuniform dose ordering, visual scoring, and unestablished observer reliability limit the strength of the morphological interpretation.

The generally dose-related pattern observed through 25 mg/kg differs from some organ-specific findings reported by Cassol et al., in which CBD effects varied with dose, tissue, and acute versus extended administration [34]. Differences in CLP severity, needle gauge, number of punctures, supportive care, sex, treatment timing, formulation, and sampling time can readily change both cytokine and oxidative trajectories [10,11,36]. CBD also has complex concentration-dependent immunological effects [30]. These sources of heterogeneity justify reporting the present regimen precisely and discourage extrapolation from a single acute dose–response curve.

The design integrates three CBD doses, a polymicrobial CLP model, and host-response and culture outcomes at a common early time point. Its limitations are substantial: pragmatic sample size without a confirmatory primary endpoint, a single 10 h endpoint, male animals only, unavailable pharmacokinetic and dedicated toxicity data, and no survival or direct target-engagement assessment. The intentional omission of postoperative analgesia to prevent interference with inflammatory endpoints, along with the absence of supportive fluid resuscitation and antibiotic therapy, represents a translational limitation of this acute exploratory model. Histological scoring was visual, semiquantitative, and performed by a single observer; therefore, observer reliability was not formally assessed, which represents a methodological limitation. Culture summaries depend on unvalidated handling of non-detects. Antibiotics and protocolized fluids were not administered; therefore, this experiment does not model standard-of-care-treated sepsis and cannot establish CBD benefit as an adjunct to clinical treatment. Studies incorporating standard sepsis care, both sexes, serial measurements, verified analgesia, survival, safety, pharmacokinetics, and validated histological scoring are needed before clinical testing can be justified from this dataset. Testing many outcomes increases the chance that some statistically significant findings occur by chance. Tukey adjustment reduces this risk for pairwise comparisons within each outcome but does not address the full panel of outcomes. The concordant directions across several domains support further investigation, yet correlated endpoints do not provide independent confirmation and cannot remove this limitation. The results should guide prospectively powered preclinical studies rather than be treated as definitive evidence of efficacy or sufficient justification for a clinical sepsis trial.

## 4. Materials and Methods

### 4.1. Animals and Experimental Groups

A total of 48 adult male Wistar albino rats (aged 10–12 weeks, weighing 250–300 g) were obtained from the Experimental Animal Laboratory. Animals were housed in standard polycarbonate cages (4 rats per cage) under controlled environmental conditions (temperature: 22 ± 2 °C; relative humidity: 50–55%; 12-h light/dark cycle) with ad libitum access to standard commercial rodent chow (Bil-Yem Gıda Tarım San. Tic. Ltd. Şti., Ankara, Türkiye) and filtered tap water. The animals were acclimatized to the laboratory environment for 7 days prior to experimentation. No formal a priori power calculation was performed. The predefined experimental design included eight animals per group across six independent groups (48 animals in total). This group size was selected for this exploratory dose-ranging study while balancing the need for between-group comparisons with the principles of reduction in animal experimentation. Accordingly, the findings should be interpreted as exploratory and confirmed in prospectively powered studies. Following baseline body-weight measurements, animals were allocated to six experimental groups (*n* = 8 per group) using a computer-generated randomization sequence: The sample size was a pragmatic choice for a six-group exploratory dose-ranging experiment, rather than a calculation based on a prespecified effect size, variance, or statistical power for a primary endpoint. No confirmatory primary endpoint or endpoint-specific power target was documented. Equal group sizes facilitated comparable sampling across the three doses and the non-septic and septic reference groups, but this does not demonstrate that eight animals per group were sufficient for every outcome. Post hoc observed power was not used to justify the design.

Control group (C): Animals underwent no surgical intervention or sepsis induction and received an equivalent volume of the vehicle solution. This group was used to establish healthy baseline values while controlling for any effects associated with vehicle administration.

Sham group (S): Animals underwent laparotomy and cecal manipulation without ligation or puncture and received vehicle only. This group was included to account for the non-specific effects of anesthesia, laparotomy, and surgical manipulation.

Sepsis group (CLP): Sepsis was induced using the cecal ligation and puncture procedure, and the animals received vehicle only. This group served as the untreated septic control.

CLP + CBD 2.5 mg/kg group: Following CLP-induced sepsis, animals received cannabidiol (CBD) at a dose of 2.5 mg/kg administered intraperitoneally.

CLP + CBD 5 mg/kg group: Following CLP-induced sepsis, animals received CBD at a dose of 5 mg/kg administered intraperitoneally.

CLP + CBD 25 mg/kg group: Following CLP-induced sepsis, animals received CBD at a dose of 25 mg/kg administered intraperitoneally.

**Figure 3:**
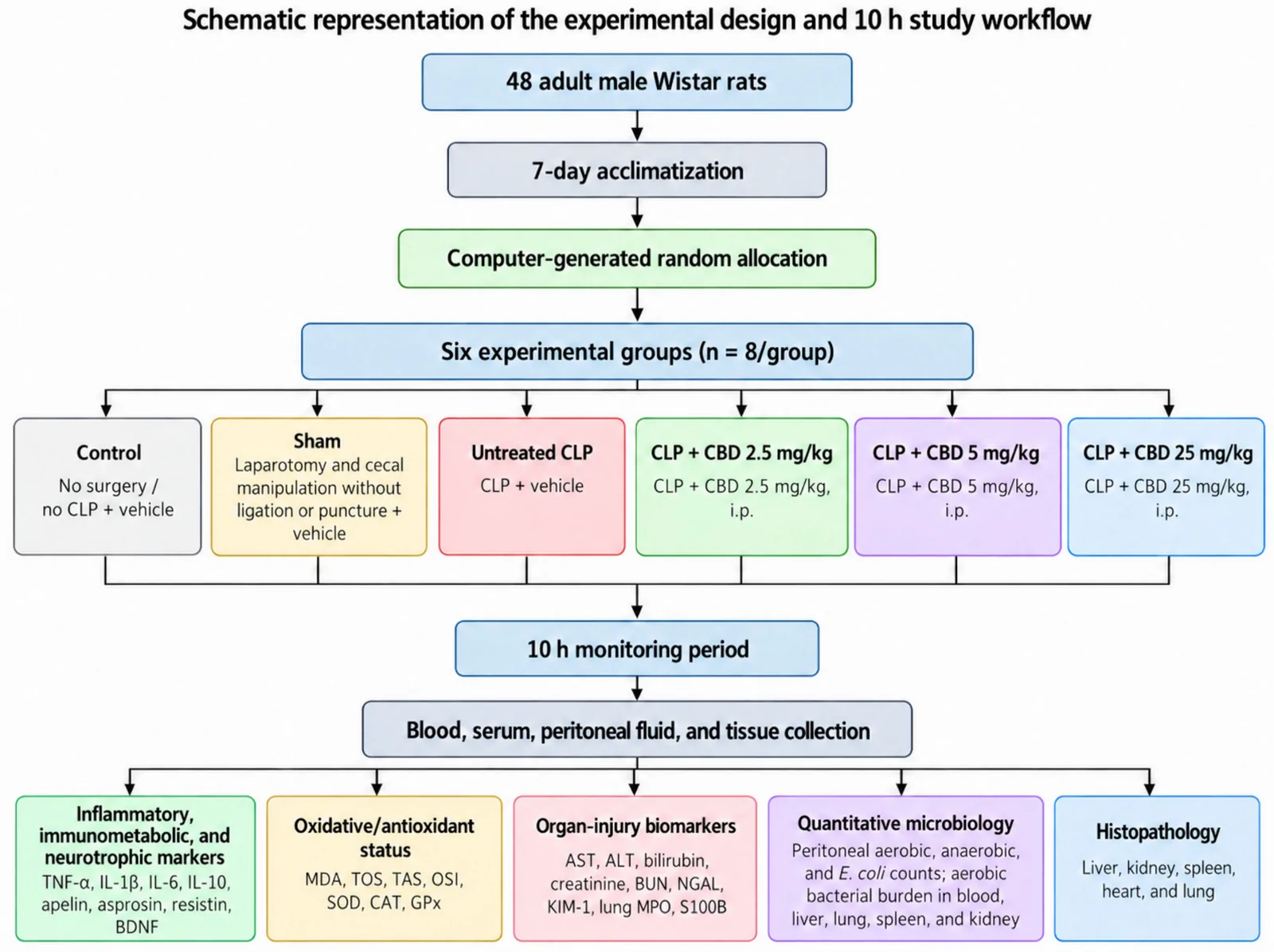
Schematic representation of the experimental design and 10 h study workflow. Forty-eight adult male Wistar rats were acclimatized for 7 days and randomly allocated to six groups (*n* = 8/group): Control, Sham, Untreated CLP, CLP + CBD 2.5 mg/kg, CLP + CBD 5 mg/kg, and CLP + CBD 25 mg/kg. Control, Sham, and Untreated CLP animals received the vehicle solution, whereas CBD-treated groups received the corresponding CBD dose intraperitoneally following CLP. After the 10 h monitoring period, blood, serum, peritoneal fluid, and tissue samples were collected for inflammatory, immunometabolic, neurotrophic, oxidative/antioxidant, biochemical organ-injury, microbiological, and histopathological assessments.

The individual animal was the experimental unit for every quantitative analysis. Predefined humane endpoints included severe hypothermia unresponsive to warming, complete lack of responsiveness or mobility, and severe dyspnea; all animals completed the 10 h observation period without reaching a terminal threshold. Investigators conducting biochemical, microbiological, and histopathological assessments were blinded to allocation. Reporting was reviewed against ARRIVE 2.0; documentation gaps are identified below. The study was approved by the Local Ethics Committee for Animal Experiments of Ankara Training and Research Hospital, University of Health Sciences, Republic of Türkiye Ministry of Health (Meeting No. 0087; Decision No. 818; 16 January 2025). The use of males reduced sex-related biological variability in this exploratory dose-ranging experiment, but exclusion of females limits generalizability and is acknowledged as a study limitation. The overall experimental design and 10 h study workflow are summarized in Figure 3.

### 4.2. Cecal Ligation and Puncture

Polymicrobial sepsis was induced using an established CLP procedure [10,11,38]. Rats were anesthetized with ketamine (Ketalar, Pfizer Inc., New York, NY, USA) (50 mg/kg) and xylazine (Rompun, Bayer AG, Leverkusen, Germany) (10 mg/kg). Through an aseptically prepared midline incision, the cecum was exteriorized, ligated approximately 1 cm distal to the apex without obstructing intestinal continuity, and perforated through-and-through at four sites with a 20-gauge needle. A small amount of fecal material was extruded to verify patency, the cecum was returned, and the incision was closed in layers. Sham animals underwent laparotomy and cecal manipulation without ligation or puncture. Animals were observed continuously until recovery from anesthesia and then at regular intervals for activity, posture, respiration, responsiveness, and temperature-related signs. No antibiotics or protocolized fluid resuscitation were administered, because the experiment was designed to isolate the early biological response to CBD; this design choice limits translational relevance and precludes comparison with standard sepsis care. Ketamine (50 mg/kg) and xylazine (10 mg/kg) were used for surgical anesthesia, providing intraoperative sedation and visceral analgesia. Separate postoperative analgesics (e.g., NSAIDs or opioids) were intentionally omitted in accordance with the approved protocol to avoid confounding interference with systemic inflammatory cascades (TNF-α, IL-1β, IL-6), oxidative stress parameters, and leukocyte responses, which represented the primary endpoints of the study. Animals were maintained under close observation throughout the 10 h monitoring period and evaluated against predefined humane endpoints (severe hypothermia unresponsive to warming, total lack of mobility/responsiveness, or severe respiratory distress). No animal reached the humane endpoint threshold prior to the scheduled 10 h termination.

### 4.3. CBD Administration and Study Endpoint

Cannabidiol (CBD; crystalline solid, purity ≥98%, Cat. No. 90080; Cayman Chemical, Ann Arbor, MI, USA) was freshly formulated in a vehicle containing 2% dimethyl sulfoxide (DMSO) and 2% Tween-80 in physiological saline immediately before administration. The 2.5 and 5 mg/kg doses were selected based on a previous CLP study in rats in which CBD was administered at 2.5, 5, and 10 mg/kg [34]. The 25 mg/kg dose was included as an exploratory upper dose to determine whether the biological response extended beyond the previously investigated CLP dose range. Accordingly, this study was designed as an exploratory dose-ranging experiment and was not intended to identify an optimal, safe, or clinically applicable CBD dose. The 25 mg/kg dose extends the previous 10 mg/kg CLP range 2.5-fold and was intended to explore a wider exposure-response range. This is a dose-ranging rationale, not evidence of safety in septic rats. No separate tolerability study, plasma or tissue CBD measurement, or formal toxicity endpoint was available. Completion of the 10 h observation period cannot exclude adverse effects. Consequently, 25 mg/kg is described only as the tested dose with the most consistent short-term biological association, not as an optimal or preferred regimen.

Following cecal ligation and puncture (CLP), CBD was administered intraperitoneally at doses of 2.5, 5, or 25 mg/kg at a standard injection volume of 1 mL/kg immediately, within 15 min after surgery. Control, Sham, and Untreated CLP animals received an equivalent volume of the corresponding vehicle under identical handling conditions.

The 10 h endpoint was selected a priori to capture the early inflammatory, oxidative, and organ-injury responses associated with this CLP protocol before substantial attrition could affect cross-group tissue sampling. The experiment was not designed to assess disease progression, long-term survival, or durable recovery. At 10 h, animals were deeply anesthetized, blood was collected intracardially, and euthanasia was completed while the animals remained under deep anesthesia, in accordance with the approved experimental protocol. Liver, kidney, spleen, heart, lung, and brain samples, together with peritoneal fluid, were collected in a fixed order using sterile instruments where appropriate.

### 4.4. Biochemical and Immunoassay Measurements

Serum AST, ALT, total bilirubin, creatinine, and BUN were quantified on a Roche Cobas c501 analyzer (Roche Diagnostics, Mannheim, Germany). Rat-specific immunoassays were used for IL-6 (BT Lab, E0135Ra), TNF-α (BT Lab, E0764Ra), IL-10 (BT Lab, E0108Ra), IL-1β (BT Lab, E0119Ra), NGAL (BT Lab, E0762Ra), KIM-1 (BT Lab, E0549Ra), S100B (BT Lab, E1360Ra), resistin (BT Lab, E0211Ra), asprosin (FineTest, ER1944), apelin (YL Biont, YLA0312RA), BDNF (YL Biont, YLA0314RA), and lung MPO (BT Lab, E0574Ra). Lung MPO was measured in lung tissue homogenates using the corresponding according to the manufacturer’s instructions. MPO values were normalized to tissue protein content and expressed as ng/g protein. Samples, standards, and quality controls were processed according to the manufacturers’ instructions, and optical densities were read at 450 nm using a microplate reader Multiskan SkyHigh Microplate Spectrophotometer, Cat. No. A51119500C; Thermo Fisher Scientific, Waltham, MA, USA).

Serum AST, ALT, total bilirubin, creatinine, and BUN were quantified on a Roche Cobas c501 analyzer (Roche Diagnostics, Mannheim, Germany). Commercially available Rat-specific immunoassays (ELISA) kits were used for IL-6 (Cat. No. E0135Ra; Bioassay Technology Laboratory [BT Lab], Shanghai, China), TNF-α (Cat. No. E0764Ra; BT Lab, Shanghai, China), IL-10 (Cat. No. E0108Ra; BT Lab, Shanghai, China), IL-1β (Cat. No. E0119Ra; BT Lab, Shanghai, China), NGAL (Cat. No. E0762Ra; BT Lab, Shanghai, China), KIM-1 (Cat. No. E0549Ra; BT Lab, Shanghai, China), S100B (Cat. No. E1360Ra; BT Lab, Shanghai, China), resistin (Cat. No. E0211Ra; BT Lab, Shanghai, China), asprosin (Cat. No. ER1944; Wuhan Fine Biotech Co., Ltd. [FineTest], Wuhan, China), apelin (Cat. No. YLA0312RA; Shanghai YL Biotech Co., Ltd. [YL Biont], Shanghai, China), BDNF (Cat. No. YLA0314RA; YL Biont, Shanghai, China), and lung MPO (Cat. No. E0574Ra; BT Lab, Shanghai, China). Lung MPO was measured in lung tissue homogenates using the corresponding rat-specific ELISA kit (Bioassay Technology Laboratory [BT Lab], Shanghai, China) according to the manufacturer’s instructions. MPO values were normalized to tissue protein content and expressed as ng/g protein. Samples, standards, and quality controls were processed according to the manufacturers’ instructions, and optical densities were read at 450 nm using a microplate reader (Multiskan SkyHigh Microplate Spectrophotometer, Cat. No. A51119500C; Thermo Fisher Scientific, Waltham, MA, USA).

All standards and serum/tissue samples were assayed in duplicate. Serum and tissue homogenate samples were assayed directly or diluted in assay buffer when concentrations exceeded the validated standard-curve range. Standard curves were constructed using a four-parameter logistic (4-PL) curve fit. According to the manufacturers’ performance specifications, the intra-assay and inter-assay coefficients of variation for the ELISA kits used were within the stated acceptable ranges. Values outside the validated range were not extrapolated without documented dilution and repeat measurement.

Whole blood was collected into standard non-anticoagulant serum separator tubes and allowed to clot for 30 min at room temperature before centrifugation at 3000× *g* for 15 min at 4 °C (Allegra X-30R Centrifuge; Beckman Coulter, Brea, CA, USA). The resulting serum was promptly separated, aliquoted into sterile microcentrifuge tubes to prevent repeated freeze–thaw cycles, and stored at −80 °C until biochemical analysis. Excised liver, kidney, spleen, heart, lung, and brain tissues were immediately rinsed with ice-cold phosphate-buffered saline (PBS, pH 7.4; Sigma-Aldrich, St. Louis, MO, USA) to remove residual blood, blotted dry, weighed where appropriate, and allocated to the biochemical, microbiological, or histopathological analyses specified for each tissue. Tissue aliquots for biochemical assays were homogenized on ice in 9 volumes (1:10 *w*/*v*) of cold PBS (pH 7.4) using a mechanical homogenizer (TissueLyser II; Qiagen, Hilden, Germany) and centrifuged at 12,000× *g* for 15 min at 4 °C; the clarified supernatants were collected, aliquoted, and preserved at −80 °C. All samples underwent only a single freeze–thaw cycle prior to analytical testing. Microbiological aliquots were transferred aseptically into sterile preweighed tubes and processed immediately. Histology aliquots were fixed in 10% neutral-buffered formalin (Sigma-Aldrich, St. Louis, MO, USA) for 48 h at room temperature.

### 4.5. Oxidative and Antioxidant Status

Malondialdehyde (MDA) was measured in liver tissue homogenates using a rat-specific ELISA kit (Cat. No. E0156Ra; Bioassay Technology Laboratory [BT Lab], Shanghai, China) according to the manufacturer’s instructions. Superoxide dismutase (SOD), catalase (CAT), and glutathione peroxidase (GPx) were also determined in liver tissue homogenates. Total protein concentrations in liver homogenates were measured using the bicinchoninic acid (BCA) protein assay kit (Pierce, Thermo Fisher Scientific, Waltham, MA, USA), and tissue MDA, SOD, CAT, and GPx values were normalized to total protein content and expressed as nmol/mg protein for MDA and ng/mg protein for SOD, CAT, and GPx. Serum total oxidant status (TOS) and total antioxidant status (TAS) were measured using commercially available colorimetric assay kits (Rel Assay Diagnostics, Gaziantep, Türkiye) according to the manufacturer’s instructions. TOS results were expressed as μmol H_2_O_2_ equivalent/L, whereas TAS results were expressed as mmol Trolox equivalent/L. The oxidative stress index (OSI) was calculated after unit harmonization using the following formula: OSI (arbitrary unit) = [TOS (μmol H_2_O_2_ Eq/L)/TAS (μmol Trolox Eq/L)] × 100, with TAS values converted from mmol/L to μmol/L before calculation. The BT Lab kits (Cat. No. E0869Ra for CAT, E1172Ra for GPx, and E0168Ra for SOD; Bioassay Technology Laboratory [BT Lab], Shanghai, China) were used according to the manufacturer’s instructions to quantify immunoreactive antioxidant-enzyme protein concentrations in tissue homogenates. These ELISA-based measurements represent immunoreactive protein concentrations rather than catalytic enzyme activity. Values were normalized to total protein content determined using the BCA assay and expressed as ng/mg protein.

### 4.6. Quantitative Microbiological Analysis

Peritoneal fluid and blood were collected aseptically, and liver, lung, spleen, and kidney specimens were aseptically weighed and homogenized in sterile phosphate-buffered saline (1:10 *w*/*v*). Serial ten-fold dilutions (10^−1^–10^−6^) were prepared in sterile saline, and 100 μL aliquots were plated in duplicate. Total aerobic bacterial counts were determined on Brain Heart Infusion (BHI) agar (Merck, Darmstadt, Germany) following aerobic incubation at 37 °C for 24–48 h. Total anaerobic bacterial counts were determined on Schaedler Anaerobe agar (Oxoid, Basingstoke, UK) supplemented with 5% sheep blood and incubated anaerobically at 37 °C for 48–72 h. Escherichia coli counts were determined on Eosin Methylene Blue (EMB) agar (Merck KGaA, Darmstadt, Germany) following incubation at 37 °C for 24 h, with presumptive colonies confirmed by Gram staining and standard biochemical tests. Peritoneal fluid was assessed for aerobic, anaerobic, and E. coli counts. Blood, liver, lung, spleen, and kidney bacterial burdens reported in Table 4 represent total aerobic bacterial counts determined on BHI agar. Plates containing 30–300 colony-forming units (CFU) were used for enumeration. Results were expressed as log_10_ CFU/mL for peritoneal fluid and blood and as log_10_ CFU/g for tissue samples. The lower detection limit was 1.0 log_10_ CFU/mL for fluid samples and 1.0 log_10_ CFU/g for tissue samples. Samples with no culture-recoverable colonies above the assay detection limit were assigned a value of 0 for descriptive tabulation and group-level summary calculations. Consequently, group mean values could fall below the nominal detection limit when a group contained both culture-negative and culture-positive animals. Samples yielding no recoverable bacterial colonies above the assay limit of detection (LOD: 1.0 log_10_ CFU/mL for fluids and 1.0 log_10_ CFU/g for tissues) were assigned a value of 0 solely as a non-detect category for exploratory data tabulation, rather than representing an absolute mathematical log_10_(0) or definitive tissue sterility. Because group summaries encompass both culture-positive animals and non-detect observations, group mean values near the detection threshold should be interpreted with caution. Consequently, bacterial load comparisons are reported as exploratory indices of recoverable microbial burden under the defined experimental conditions, and they do not imply direct in vivo bactericidal efficacy of CBD.

### 4.7. Histopathology

Liver, kidney, spleen, heart, and lung specimens were fixed for 48 h in 10% neutral-buffered formalin. Following fixation, the tissues were processed through graded alcohol and xylene series using an automated tissue processor (Leica TP1020, Leica Microsystems, Wetzlar, Germany) and embedded in paraffin. Sections of 5 µm thickness were obtained using a rotary microtome (Shandon AS325; Thermo Fisher Scientific, Waltham, MA, USA) and stained with hematoxylin and eosin (H&E). Histopathological examinations and representative image acquisition were performed using an Olympus BX51 light microscope equipped with a DP70 digital camera (Olympus Corporation, Tokyo, Japan).

Ten representative microscopic fields per tissue section were evaluated at 100× magnification. Histopathological alterations were classified into three domains: (i) degeneration and necrosis, including hydropic, vacuolar, or parenchymatous degeneration; (ii) inflammatory changes, characterized by interstitial infiltration of neutrophils, lymphocytes, and macrophages; and (iii) vascular changes, principally hyperemia and capillary congestion. For each lesion domain, the proportion of the tissue area affected within each examined field was visually estimated as a percentage (0–100%). The mean percentage across the 10 evaluated fields was then calculated for each animal and used as the individual histopathological lesion score. For descriptive interpretation, lesion extent was categorized as absent (0%), very low (1–10%), low (11–25%), low-to-moderate (26–40%), moderate (41–60%), severe (61–80%), or very severe (81–100%).

Histopathological evaluation included all six experimental groups: Control, Sham, Untreated CLP, CLP + CBD 2.5 mg/kg, CLP + CBD 5 mg/kg, and CLP + CBD 25 mg/kg. Eight animals from each group were included in the histopathological assessment, consistent with the overall experimental design. No missing histopathological values were imputed. Histopathological findings were evaluated as a complementary morphological component of the study and interpreted together with the biochemical, inflammatory, oxidative, and microbiological findings. As stated in Section 4.1, histopathological evaluations were performed by a single experienced investigator who was strictly blinded to the experimental group allocations. Because all assessments were carried out by a single observer under identical standardized microscopy conditions across ten randomly selected microscopic fields per organ, inter-observer reliability analysis was not applicable. The visually estimated scores are presented as descriptive, semiquantitative histological outcomes, and this methodology has been explicitly acknowledged as an exploratory limitation of the study.

### 4.8. Statistical Analysis

Continuous biochemical and microbiological outcomes were summarized as mean ± standard deviation. The individual animal was considered the statistical unit (*n* = 8 per group). For each endpoint, the six independent experimental groups were compared using one-way analysis of variance (ANOVA), followed by Tukey-adjusted pairwise comparisons. Residual normality and homogeneity of variance were evaluated using the Shapiro–Wilk and Levene tests, respectively. All tests were two-sided, and *p* < 0.05 was considered statistically significant. Because the microbiological datasets included culture-negative observations recorded as zero, statistical comparisons of bacterial-burden outcomes were interpreted as exploratory and considered together with the magnitude and consistency of the observed group differences. Tukey adjustment applies to pairwise comparisons within each endpoint and does not control false-positive findings across the complete endpoint panel. No study-wide family-wise error or false-discovery-rate adjustment was applied. Nominal *p* values therefore indicate exploratory associations, not confirmatory efficacy.

Histopathology was analyzed descriptively at the individual-animal level (*n* = 8/group); the ten fields per section were not treated as independent biological replicates. Table 5 summarizes means ± SD by group, organ, and lesion domain, and Figure 2 displays the mean lesion percentages. No missing histopathological scores were imputed and no histopathological efficacy test is claimed.

## 5. Conclusions

In this exploratory 10 h rat CLP experiment, CBD exposure was associated with changes in inflammatory, redox, biochemical, immunometabolic, neurotrophic, microbial, and morphological outcomes. The 25 mg/kg regimen showed the most consistent short-term biological association among the tested doses, without establishing optimal dosing, safety, durable organ recovery, or clinical benefit. Interpretation of the microbial findings requires validation of animal-level CFU data and detection-limit handling. These observations support further preclinical studies with standard sepsis care, documented analgesia, both sexes, prospective power calculations, pharmacokinetics, toxicity and survival endpoints, and direct mechanistic measurements.

## Figures and Tables

**Table 1:** Hepatic, renal, pulmonary, and cerebral injury biomarkers.

Parameter	Organ f2.	Control	Sham	CLP	CBD 2.5	CBD 5	CBD 25	*p*
AST (U/L)	Liver	82.4 ± 6.8 ^d^	86.1 ± 7.2 ^d^	248.5 ± 18.6 ^a^	184.2 ± 14.3 ^b^	128.7 ± 11.5 ^c^	98.4 ± 8.1 ^d^	<0.001
ALT (U/L)		38.6 ± 3.4 ^d^	41.2 ± 4.1 ^d^	136.4 ± 11.2 ^a^	98.5 ± 8.7 ^b^	64.3 ± 5.9 ^c^	46.2 ± 4.5 ^d^	<0.001
Bilirubin (mg/dL)		0.24 ± 0.03 ^d^	0.26 ± 0.04 ^d^	0.92 ± 0.08 ^a^	0.68 ± 0.06 ^b^	0.45 ± 0.05 ^c^	0.31 ± 0.04 ^d^	<0.001
Creatinine (mg/dL)	Kidney	0.42 ± 0.05 ^d^	0.45 ± 0.06 ^d^	1.68 ± 0.14 ^a^	1.24 ± 0.11 ^b^	0.82 ± 0.08 ^c^	0.54 ± 0.06 ^d^	<0.001
BUN (mg/dL)		18.5 ± 1.9 ^a^	20.1 ± 2.2 ^ae^	68.4 ± 5.7 ^b^	51.2 ± 4.3 ^c^	34.6 ± 3.1 ^d^	23.8 ± 2.4 ^e^	<0.001
NGAL (ng/mL)		14.2 ± 1.5 ^d^	15.8 ± 1.7 ^d^	74.6 ± 6.2 ^a^	54.1 ± 4.8 ^b^	32.5 ± 3.2 ^c^	19.4 ± 2.1 ^d^	<0.001
KIM-1 (pg/mL)		112.5 ± 10.4 ^d^	118.3 ± 11.2 ^d^	486.2 ± 36.5 ^a^	358.4 ± 28.1 ^b^	224.6 ± 19.8 ^c^	145.2 ± 13.6 ^d^	<0.001
Lung MPO (ng/g protein)	Lung	0.85 ± 0.09 ^d^	0.92 ± 0.11 ^d^	4.12 ± 0.38 ^a^	2.95 ± 0.26 ^b^	1.74 ± 0.18 ^c^	1.12 ± 0.12 ^d^	<0.001
S100B (pg/mL)	Brain-related marker	42.3 ± 4.1 ^d^	45.8 ± 4.6 ^d^	196.4 ± 16.2 ^a^	142.1 ± 12.5 ^b^	88.6 ± 8.4 ^c^	53.7 ± 5.2 ^d^	<0.001

Values are mean ± SD (*n* = 8/group). Omnibus *p* values are from one-way ANOVA. CBD doses are expressed as mg/kg. BUN, blood urea nitrogen; KIM-1, kidney injury molecule-1; MPO, myeloperoxidase; NGAL, neutrophil gelatinase-associated lipocalin. Means without a common lowercase superscript differ at Tukey-adjusted *p* < 0.05. Lowercase letters summarize the original within-endpoint Tukey comparisons; they do not denote a study-wide multiplicity correction. The Results text emphasizes Untreated CLP versus Control and each CBD dose versus Untreated CLP. The organ function column groups markers by clinical interpretation; AST is not liver-specific, NGAL and KIM-1 are injury markers, lung MPO reflects neutrophil-associated inflammation, and S100B alone does not measure cerebral function.

**Table 2:** Serum inflammatory cytokines, metabolic peptides, and BDNF.

Parameter	Control	Sham	CLP	CBD 2.5	CBD 5	CBD 25	*p*
TNF-α (pg/mL)	24.5 ± 2.8 ^a^	27.1 ± 3.1 ^ae^	184.6 ± 15.2 ^b^	132.4 ± 11.6 ^c^	76.8 ± 7.2 ^d^	39.2 ± 4.1 ^e^	<0.001
IL-1β (pg/mL)	18.2 ± 2.1 ^d^	20.4 ± 2.4 ^d^	142.8 ± 12.7 ^a^	96.5 ± 8.9 ^b^	52.1 ± 5.4 ^c^	28.4 ± 3.2 ^d^	<0.001
IL-6 (pg/mL)	35.4 ± 3.8 ^a^	39.6 ± 4.2 ^ae^	295.4 ± 24.1 ^b^	205.8 ± 17.5 ^c^	114.2 ± 10.8 ^d^	58.6 ± 5.9 ^e^	<0.001
IL-10 (pg/mL)	68.4 ± 6.2 ^c^	65.1 ± 5.9 ^cd^	28.6 ± 3.1 ^a^	42.5 ± 4.3 ^b^	58.9 ± 5.6 ^d^	64.2 ± 6.1 ^cd^	<0.001
Apelin (ng/mL)	4.85 ± 0.42 ^c^	4.62 ± 0.39 ^c^	1.54 ± 0.16 ^a^	2.68 ± 0.25 ^b^	3.92 ± 0.34 ^d^	4.41 ± 0.38 ^cd^	<0.001
Asprosin (ng/mL)	3.12 ± 0.31 ^d^	3.35 ± 0.34 ^d^	11.84 ± 1.05 ^a^	8.42 ± 0.76 ^b^	5.61 ± 0.52 ^c^	3.89 ± 0.38 ^d^	<0.001
Resistin (ng/mL)	2.45 ± 0.26 ^d^	2.68 ± 0.28 ^d^	9.75 ± 0.88 ^a^	6.92 ± 0.64 ^b^	4.58 ± 0.45 ^c^	3.15 ± 0.32 ^d^	<0.001
BDNF (pg/mL)	845.2 ± 65.4 ^d^	820.6 ± 61.2 ^d^	295.8 ± 28.4 ^a^	465.3 ± 42.1 ^b^	684.5 ± 58.7 ^c^	795.1 ± 62.3 ^d^	<0.001

Values are mean ± SD (*n* = 8/group). Omnibus *p* values are from one-way ANOVA. BDNF, brain-derived neurotrophic factor; IL, interleukin; TNF-α, tumor necrosis factor-α. Means without a common lowercase superscript differ at Tukey-adjusted *p* < 0.05. Lowercase letters summarize the original within-endpoint Tukey comparisons; they do not denote a study-wide multiplicity correction. The Results text emphasizes Untreated CLP versus Control and each CBD dose versus Untreated CLP.

**Table 3:** Serum oxidative-status indices and liver tissue oxidative and antioxidant parameters.

Parameter	Control	Sham	CLP	CBD 2.5	CBD 5	CBD 25	*p*
MDA (nmol/mg protein)	1.82 ± 0.18 ^d^	1.95 ± 0.21 ^d^	6.84 ± 0.58 ^a^	4.92 ± 0.44 ^b^	3.15 ± 0.29 ^c^	2.24 ± 0.22 ^d^	<0.001
TOS (μmol H_2_O_2_ Eq/L)	8.45 ± 0.82 ^d^	8.92 ± 0.88 ^d^	28.65 ± 2.45 ^a^	20.42 ± 1.85 ^b^	14.18 ± 1.32 ^c^	10.15 ± 0.95 ^d^	<0.001
TAS (mmol Trolox Eq/L)	1.75 ± 0.16 ^c^	1.68 ± 0.15 ^c^	0.72 ± 0.08 ^a^	1.08 ± 0.11 ^b^	1.45 ± 0.14 ^d^	1.62 ± 0.15 ^cd^	<0.001
OSI (arbitrary unit)	0.48 ± 0.05 ^d^	0.53 ± 0.06 ^d^	3.98 ± 0.38 ^a^	1.89 ± 0.19 ^b^	0.98 ± 0.10 ^c^	0.63 ± 0.07 ^d^	<0.001
SOD (ng/mg protein)	45.8 ± 4.2 ^c^	43.5 ± 3.9 ^c^	16.4 ± 1.8 ^a^	26.2 ± 2.5 ^b^	36.8 ± 3.4 ^d^	41.5 ± 3.8 ^cd^	<0.001
CAT (ng/mg protein)	32.4 ± 3.1 ^d^	30.8 ± 2.9 ^d^	11.2 ± 1.2 ^a^	18.5 ± 1.7 ^b^	25.6 ± 2.4 ^c^	29.8 ± 2.7 ^d^	<0.001
GPx (ng/mg protein)	28.6 ± 2.7 ^d^	27.2 ± 2.5 ^d^	9.8 ± 1.1 ^a^	16.4 ± 1.6 ^b^	22.8 ± 2.1 ^c^	26.5 ± 2.4 ^d^	<0.001

Values are mean ± SD (*n* = 8/group). Omnibus *p* values are from one-way ANOVA. TOS, TAS, and OSI represent serum measurements, whereas MDA, SOD, CAT, and GPx were measured in liver tissue homogenates and normalized to total protein content. CAT, catalase; GPx, glutathione peroxidase; MDA, malondialdehyde; OSI, oxidative stress index; SOD, superoxide dismutase; TAS, total antioxidant status; TOS, total oxidant status. Means without a common lowercase superscript differ at Tukey-adjusted *p* < 0.05. Lowercase letters summarize the original within-endpoint Tukey comparisons; they do not denote a study-wide multiplicity correction. The Results text emphasizes Untreated CLP versus Control and each CBD dose versus Untreated CLP.

**Table 4:** Quantitative bacterial load in fluid, blood, and tissues.

Parameter	Control	Sham	CLP	CBD 2.5	CBD 5	CBD 25	*p*
Peritoneal aerobic (log_10_ CFU/mL)	0.00 ± 0.00 ^a^	0.00 ± 0.00 ^a^	6.85 ± 0.52 ^b^	5.12 ± 0.41 ^c^	3.64 ± 0.35 ^d^	2.15 ± 0.24 ^e^	<0.001
Peritoneal anaerobic (log_10_ CFU/mL)	0.00 ± 0.00 ^a^	0.00 ± 0.00 ^a^	7.42 ± 0.61 ^b^	5.86 ± 0.48 ^c^	4.15 ± 0.39 ^d^	2.68 ± 0.29 ^e^	<0.001
Peritoneal *Escherichia coli* (log_10_ CFU/mL)	0.00 ± 0.00 ^a^	0.00 ± 0.00 ^a^	5.94 ± 0.46 ^b^	4.38 ± 0.37 ^c^	2.85 ± 0.28 ^d^	1.42 ± 0.18 ^e^	<0.001
Blood aerobic (log_10_ CFU/mL)	0.00 ± 0.00 ^a^	0.00 ± 0.00 ^a^	4.68 ± 0.42 ^b^	3.24 ± 0.31 ^c^	1.85 ± 0.22 ^d^	0.65 ± 0.12 ^e^	<0.001
Liver aerobic (log_10_ CFU/g)	0.00 ± 0.00 ^a^	0.00 ± 0.00 ^a^	5.24 ± 0.45 ^b^	3.78 ± 0.34 ^c^	2.41 ± 0.26 ^d^	1.18 ± 0.15 ^e^	<0.001
Lung aerobic (log_10_ CFU/g)	0.00 ± 0.00 ^a^	0.00 ± 0.00 ^a^	4.82 ± 0.39 ^b^	3.45 ± 0.32 ^c^	2.12 ± 0.24 ^d^	0.95 ± 0.14 ^e^	<0.001
Spleen aerobic (log_10_ CFU/g)	0.00 ± 0.00 ^a^	0.00 ± 0.00 ^a^	5.62 ± 0.48 ^b^	4.15 ± 0.38 ^c^	2.74 ± 0.29 ^d^	1.36 ± 0.17 ^e^	<0.001
Kidney aerobic (log_10_ CFU/g)	0.00 ± 0.00 ^a^	0.00 ± 0.00 ^a^	4.15 ± 0.36 ^b^	2.86 ± 0.27 ^c^	1.65 ± 0.19 ^d^	0.52 ± 0.10 ^e^	<0.001

Values are presented as mean ± SD (*n* = 8/group). Omnibus *p* values were obtained by one-way ANOVA. Peritoneal aerobic, anaerobic, and *Escherichia coli* counts are expressed as log_10_ CFU/mL. Blood values represent total aerobic bacterial counts and are expressed as log_10_ CFU/mL, whereas liver, lung, spleen, and kidney values represent total aerobic bacterial counts and are expressed as log_10_ CFU/g. Zero values are coded non-detects, not measured log_10_ concentrations or proof of sterility. Because group summaries included both culture-negative and culture-positive animals, mean values could fall below the nominal detection limit. Statistical comparisons of these microbiological outcomes should therefore be interpreted as exploratory. Groups that do not share a lowercase superscript differ significantly at Tukey-adjusted *p* < 0.05. Lowercase letters summarize the original within-endpoint Tukey comparisons; they do not denote a study-wide multiplicity correction. The Results text emphasizes Untreated CLP versus Control and each CBD dose versus Untreated CLP.

**Table 5:** Group mean histopathological lesion scores as visually estimated affected tissue area (%).

Organ	Lesion Domain	Control	Sham	Untreated CLP	CBD 2.5	CBD 5	CBD 25
Liver	Degeneration and necrosis	0.0 ± 0.0	16.8 ± 7.0	51.8 ± 10.3	27.2 ± 16.9	30.0 ± 16.0	18.9 ± 12.2
Kidney	Degeneration and necrosis	0.0 ± 0.0	6.0 ± 4.1	54.2 ± 8.8	21.2 ± 8.6	13.1 ± 15.3	12.2 ± 8.6
Spleen	Degeneration and necrosis	0.0 ± 0.0	0.0 ± 0.0	26.5 ± 5.5	0.0 ± 0.0	0.0 ± 0.0	0.0 ± 0.0
Heart	Degeneration and necrosis	0.0 ± 0.0	6.8 ± 4.3	35.8 ± 6.7	4.9 ± 4.3	6.5 ± 8.3	7.4 ± 3.6
Lung	Degeneration and necrosis	0.0 ± 0.0	1.8 ± 3.4	54.5 ± 9.0	7.0 ± 3.3	4.9 ± 8.5	0.0 ± 0.0
Liver	Inflammation	0.0 ± 0.0	0.0 ± 0.0	35.9 ± 7.1	0.0 ± 0.0	0.0 ± 0.0	0.0 ± 0.0
Kidney	Inflammation	0.0 ± 0.0	4.4 ± 3.8	35.6 ± 6.9	1.6 ± 3.1	0.0 ± 0.0	0.0 ± 0.0
Spleen	Inflammation	4.6 ± 5.0	8.8 ± 1.0	35.8 ± 7.3	9.4 ± 0.9	6.6 ± 4.2	9.2 ± 10.4
Heart	Inflammation	0.0 ± 0.0	0.0 ± 0.0	26.6 ± 5.4	0.0 ± 0.0	0.0 ± 0.0	0.0 ± 0.0
Lung	Inflammation	0.0 ± 0.0	5.6 ± 4.0	54.5 ± 9.6	13.9 ± 10.3	13.9 ± 12.4	11.1 ± 5.7
Liver	Vascular changes	3.4 ± 4.7	11.4 ± 5.2	44.8 ± 9.4	7.4 ± 3.3	5.5 ± 8.6	7.2 ± 3.4
Kidney	Vascular changes	3.1 ± 4.4	1.9 ± 2.6	33.8 ± 8.2	2.0 ± 2.8	2.1 ± 4.0	0.0 ± 0.0
Spleen	Vascular changes	0.0 ± 0.0	3.6 ± 4.1	26.5 ± 4.7	1.9 ± 3.7	0.0 ± 0.0	0.0 ± 0.0
Heart	Vascular changes	5.9 ± 4.9	6.6 ± 4.4	35.5 ± 7.2	4.0 ± 3.5	3.8 ± 4.3	0.0 ± 0.0
Lung	Vascular changes	5.2 ± 4.6	3.9 ± 3.4	52.0 ± 10.0	10.6 ± 8.8	14.8 ± 13.8	4.4 ± 4.2

Values are mean ± SD (*n* = 8 animals/group). CBD, cannabidiol; CLP, cecal ligation and puncture; SD, standard deviation. No inferential histopathological test was performed.

## Data Availability

The original contributions presented in this study are included in the article. Further inquiries can be directed to the corresponding author.

## Abbreviations

ALT, alanine aminotransferase; AST, aspartate aminotransferase; BDNF, brain-derived neurotrophic factor; BUN, blood urea nitrogen; CAT, catalase; CBD, cannabidiol; CFU, colony-forming units; CLP, cecal ligation and puncture; GPx, glutathione peroxidase; H&E, hematoxylin and eosin; IL, interleukin; KIM-1, kidney injury molecule-1; MDA, malondialdehyde; MPO, myeloperoxidase; NGAL, neutrophil gelatinase-associated lipocalin; OSI, oxidative stress index; SOD, superoxide dismutase; TAS, total antioxidant status; TNF-α, tumor necrosis factor-α; TOS, total oxidant status.
